# Urine drug screening in women's forensic mental health services: is current practice meeting guidelines?

**DOI:** 10.1192/bjo.2021.903

**Published:** 2021-06-18

**Authors:** Jeremy Rampling, Shay-Anne Pantall, Ravinder Mann

**Affiliations:** 1 Birmingham and Solihull Mental Health NHS Foundation Trust; 2 University of Birmingham, Medical School

## Abstract

**Aims:**

To investigate adherence to Trust guidelines for urine drug screening amongst female forensic psychiatric inpatients.

**Background:**

The use of illicit substances is an important risk factor which needs to be considered in the management and treatment of forensic psychiatric patients. Research has demonstrated that a high proportion of women admitted within secure services in the UK have a history of substance use. Substance misuse amongst this population can lead to an increased risk of violence, re-offending and mental health relapse; which can pose a significant threat to the safety of other patients, staff and the public. It is therefore important that regular drug screening is carried out to minimise such risks. Ardenleigh is a blended female secure unit in Birmingham. The service has established specific substance use guidelines, outlining the need for each patient to have a personalised drug screen care plan in place. Here we present the findings of an audit completed in 2019.

**Method:**

A six month retrospective electronic case note audit for female inpatients admitted to Ardenleigh as of 1st September 2019 (n = 27). We compared drug screen care plans and frequency of urine drug screens over 6 months with the recommendations of the current service-specific Trust guidelines. Care plans should include: information regarding random drug screening; frequency of random drug screening; triggers for increased risk of substance misuse; and consequences for a positive test result to be contained within inpatient care plans.

**Result:**

Patient aged between 20 and 56 years old (median age 31). Fewer than half of inpatients (41%) had a documented random drug screen completed within the review period. In terms of care-planning, only 52% of patients had random drug screening mentioned in their care plan. 22% of patient care plans reported the actions/consequences for a positive test result. Not a single care plan mentioned how frequently patients should be being tested or potential triggers for increased risk of drug misuse amongst inpatients.

**Conclusion:**

Current practice and recording of drug screening amongst female forensic psychiatric patients is poor compared to expected standards. The lack of consistency in drug screening raises concerns regarding whether potential substance misuse amongst inpatients may be going undetected, and therefore impacting the recovery of patients. Improvements to drug screening practice should be considered in order to ensure optimal recovery and safety to patients and others.

